# Editorial for the Special Issue “Advances in Metabolic Engineering of Industrial Microorganisms”

**DOI:** 10.3390/microorganisms14061368

**Published:** 2026-06-20

**Authors:** Shuobo Shi

**Affiliations:** Beijing Advanced Innovation Center for Soft Matter Science and Engineering, College of Life Science and Technology, Beijing University of Chemical Technology, Beijing 100029, China; shishuobo@mail.buct.edu.cn

The past decade has witnessed transformative progress in metabolic engineering, driven by the convergence of synthetic biology, CRISPR-based genome editing, systems biology, and high-throughput omics technologies [1]. Traditional strain improvement methods, such as random mutagenesis and adaptive laboratory evolution, have been complemented by rational design tools that enable precise manipulation of metabolic fluxes, regulatory networks, and stress tolerance. These advances have broadened the scope of industrial microbiology from simply improving yield to achieving sophisticated control over pathway dynamics, cofactor balance, and cellular robustness under process conditions [2]. The five papers in this Special Issue exemplify these trends, each tackling a distinct challenge in industrial microbiology: from optimizing carbon and nitrogen metabolism to overcoming feedstock-related stresses and balancing primary metabolism with specialized metabolite production. As such, they not only advance fundamental knowledge but also provide actionable blueprints for industrial strain development.

Park and Lim provide a comprehensive review of the proteomics and central carbon metabolism of *Corynebacterium glutamicum* [3], a workhorse for amino acid production. By synthesizing decades of research, the authors highlight how proteomic analyses have elucidated key regulatory nodes and post-translational modifications that govern carbon flux. Importantly, they emphasize several advantages of *C. glutamicum*, such as the absence of endotoxins, the higher solubility of recombinant proteins, and its robustness under industrial conditions, and point to emerging synthetic biology tools that expand its product portfolio beyond amino acids to include biofuels, vitamins, and organic acids. This review fills a gap by connecting classical strain development with contemporary omics-driven strategies.

Liu et al. address a practical bottleneck in pullulan production, namely that high sugar concentrations impose hyperosmotic stress on *Aureobasidium pullulans* [4]. Through physiological, biochemical, and transcriptomic analyses, they demonstrate that exogenous proline (400 mg/L) acts as an effective osmoprotectant, increasing biomass by 10.8% and pullulan yield by 30% (to 174.8 g/L). Proline supplementation preserved cell integrity, upregulated key biosynthetic enzymes for pullulan (UGP, PGM, UGT), and altered the expression of genes involved in glycolysis, pyruvate metabolism, and sulfur metabolism. This work provides a low-cost, readily implementable strategy for improving polysaccharide production under high-sugar conditions, a clear example of how metabolic engineering can leverage compatible solutes to mitigate process stress.

Yuan et al. focus on the antitumor antibiotic geldanamycin produced by *Streptomyces geldanamycininus* [5]. Using a combined approach of protoplast preparation followed by UV mutagenesis, they obtained a mutant strain with a 36% higher yield (3742 μg/mL) than the initial type. Whole-genome resequencing and transcriptomics revealed that the high-yielding phenotype correlates with the upregulation of pathway genes *GdmG* and *GdmX* (1.6- and 2.4-fold) and the downregulation of fatty acid biosynthesis genes *pks12*, *pikAI*, and *pikAII* (0.25- to 0.48-fold). This integrated mutagenesis-omics workflow demonstrates how classical strain improvement can be rationally guided by molecular characterization, offering a template for enhancing other secondary metabolites.

Bontà et al. take a different angle by engineering a probiotic *Bacillus subtilis* strain that overproduces cellulases and xylanases to improve fiber degradability in dairy cattle feed [6]. Using an engineered isogenic strain (PB2OPT) that secretes 142-fold more cellulase and 5.4-fold more xylanase than the wild type, they treated eight feed ingredients in an in vitro rumen fermentation system. The high-enzyme strain increased neutral detergent fiber degradability (NDFd) by 10% and gas production by 9–12% compared to the control, with the greatest benefit observed for recalcitrant maize silage. Importantly, the bacteria were grown on a low-cost medium derived from rice straw washings, illustrating a circular economy approach. This work revealed that bacterial fibrolytic enzymes are responsible for the positive effects of *Bacillus* direct-fed microbials, bridging a long-standing mechanistic gap.

Abreu et al. explore the relationship between nitrogen metabolism, lipid accumulation, and antibiotic production in *Streptomyces coelicolor* [7]. By analyzing deletion mutants of genes involved in polyamine degradation (*glnA2*, *glnA3*, *glnA4*), protein turnover (*pup*), nitrogen regulation (*glnE*, *glnK*), and the global regulator *dasR*, they found that nitrogen limitation triggers triacylglycerol (TAG) accumulation. Notably, high TAG content often correlated with low cardiolipin (CL) content, suggesting a metabolic trade-off. Unexpectedly, the *dasR* mutant, which had a higher TAG content and a lower CL content, failed to produce the blue polyketide actinorhodin (ACT), whereas *glnA2* and *pup* mutants showed increased ACT production under phosphate limitation. The authors propose that TAG storage, CL reduction, and ACT biosynthesis all help mitigate oxidative stress arising from nitrogen limitation. This study reveals previously unrecognized connections between primary and secondary metabolism, with implications for engineering strains that co-produce lipids and antibiotics.

Collectively, these papers underscore several emerging themes that will shape the next decade of metabolic engineering. First, multi-omics integration, combining genome resequencing with transcriptomics, lipidomics, proteomics, and metabolomics, enables precise identification of causal mutations and metabolic bottlenecks, as demonstrated by Yuan et al. [5] and Abreu et al. [7]. Second, stress tolerance engineering remains a critical priority; Liu et al. [4] and Bontà et al. [6] have shown that engineering robustness against osmotic, oxidative, and feedstock-derived stresses via directed evolution of chaperones and global regulators is a high priority. Third, expanding synthetic biology toolkits (e.g., inducible promoters, CRISPRi/a, base editors) to non-model hosts such as actinomycetes, yeasts, and cyanobacteria will accelerate strain development, as noted by Park and Lim [3]. In parallel, the mechanistic insights revealed by Abreu et al. [7] can guide design-build-test-learn (DBTL) cycles by informing predictions of optimal editing targets and pathway fluxes. Looking ahead, AI-powered prediction of gene editing targets promises to further shorten DBTL cycles. For example, an AI-driven biofoundry platform recently achieved up to 90-fold improvements in enzyme substrate preference [8], dramatically accelerating metabolic pathway optimization. Finally, translation from laboratory to industrial scale must address bioreactor hydrodynamics, downstream processing, and long-term genetic stability [9]. Tackling these interconnected priorities, from molecular tool development to process engineering, will unlock the full potential of industrial microorganisms for sustainable biomanufacturing. These contributions illustrate a clear trajectory toward more predictive, resilient, and scalable metabolic engineering platforms.
